# Scaffold of Selenium Nanovectors and Honey Phytochemicals for Inhibition of *Pseudomonas aeruginosa* Quorum Sensing and Biofilm Formation

**DOI:** 10.3389/fcimb.2017.00093

**Published:** 2017-03-23

**Authors:** Prateeksha, Braj R. Singh, M. Shoeb, S. Sharma, A. H. Naqvi, Vijai K. Gupta, Brahma N. Singh

**Affiliations:** ^1^Pharmacognosy and Ethnopharmacology Division, Herbal Nanobiotechnology Lab, CSIR-National Botanical Research InstituteLucknow, India; ^2^Centre of Excellence in Materials Science (Nanomaterials), Z. H. College of Engineering and Technology, Aligarh Muslim UniversityAligarh, India; ^3^TERI-Deakin Nanobiotechnology Centre, The Energy Research InstituteNew Delhi, India; ^4^Molecular Glyco-Biotechnology Group, Discipline of Biochemistry, School of Natural Sciences, NUI GalwayGalway, Ireland; ^5^Department of Chemistry and Biotechnology, ERA Chair of Green Chemistry, School of Science, Tallinn University of TechnologyTallinn, Estonia

**Keywords:** selenium, nanovectors, honey, polyphenols, quorum sensing, *Pseudomonas aeruginosa*

## Abstract

Honey is an excellent source of polyphenolic compounds that are effective in attenuating quorum sensing (QS), a chemical process of cell-to-cell communication system used by the opportunistic pathogen *Pseudomonas aeruginosa* to regulate virulence and biofilm formation. However, lower water solubility and inadequate bioavailability remains major concerns of these therapeutic polyphenols. Its therapeutic index can be improved by using nano-carrier systems to target QS signaling potently. In the present study, we fabricated a unique drug delivery system comprising selenium nanoparticles (SeNPs; non-viral vectors) and polyphenols of honey (HP) for enhancement of anti-QS activity of HP against *P. aeruginosa* PAO1. The developed selenium nano-scaffold showed superior anti-QS activity, anti-biofilm efficacy, and anti-virulence potential in both *in-vitro* and *in-vivo* over its individual components, SeNPs and HP. LasR is inhibited by selenium nano-scaffold *in-vitro*. Using computational molecular docking studies, we have also demonstrated that the anti-virulence activity of selenium nano-scaffold is reliant on molecular binding that occurs between HP and the QS receptor LasR through hydrogen bonding and hydrophobic interactions. Our preliminary investigations with selenium-based nano-carriers hold significant promise to improve anti-virulence effectiveness of phytochemicals by enhancing effective intracellular delivery.

## Introduction

Emergence of multi-drug resistant bacteria has driven studies for disabling virulence preferentially through quorum sensing (QS) inhibition tactics instead of bactericidal and bacteriostatic strategies (Singh et al., 2009a; Defoirdt et al., 2013; Kim et al., 2015; Lidor et al., 2015; Srivastava et al., 2015; Wang et al., 2015; Yang et al., 2015). *P. aeruginosa*, a Gram-negative bacterium causes cystic fibrosis in immunocompromised patients, burn units of hospitals, and in implanted medical devices including intubation tubes and stents (Singh et al., 2012; Singh B. R. et al., 2015; Kalferstova et al., 2016). *P*. *aeruginosa* uses *N*-acyl homoserine lactones (AHL)-mediated QS signal cascade to trigger expression of dozens of virulence genes and biofilm formation when it reaches sufficient population densities (Chugani et al., 2012; Yin et al., 2016). In particular, it is lethal to cystic fibrosis patients (common genetic disorder in Caucasians) by developing mucoid in lung tissue leading to pneumonia. Opportunely, numerous plant-derived QS inhibitors have been developed and the infection rate has decreased in the past few years (Hentzer et al., 2002; Hentzer and Givskov, 2003; Singh et al., 2009b; Singh B. N. et al., 2015). However, the current anti-QS therapies often are not straightforward owing to their lower bioavailability and inadequate penetration of bacterial biofilm and thus results in significant casualties (Defoirdt et al., 2013). The development of a novel series of bio-nanomaterials for efficient drug delivery opens a new prospective to rectify these problems.

The combination of biology and nanotechnology has led to the creation of an interdisciplinary area, bio-nanotechnology that shows wide utilizations in medicine for targeted drug delivery in many diseases and disorders (de la Zerda and Gambhir, 2007; Zhu et al., 2013; Butcher et al., 2016). The basic justification is being excellent optical, magnetic, or structural physico-chemical properties that are not available with molecules or bulk solids. The outstanding performance of bio-nanomaterials opens novel possibilities for drug delivery and therapy of diseases that have traditionally been proven ineffective via conventional methods, particularly for microbial infections (Fernandes et al., 2010).

A range of nano-systems with different structure and compositions, such as metals, polymers, oxides, and semiconductors, have been fabricated to carry antimicrobial agents (de la Zerda and Gambhir, 2007). Among these nanomaterials, selenium nanoparticles (SeNPs) have garnered a great deal of attention as efficient drug carriers (Liu et al., 2012; Yu et al., 2014; Xia et al., 2015). As a special Se species, the SeNPs are also recognized because of their excellent antioxidant property and disease preventive effects (Torres et al., 2012; Liao et al., 2015). Abundant evidences also support the better biocompatibility, bio-efficacy and lower toxicity of the SeNPs by comparing with inorganic and organic seleno-compounds (Husen and Siddiqi, 2014). Many studies have revealed that the SeNPs could inhibit the growth of pathogenic microorganisms (Tran and Webster, 2011; Khiralla and El-Deeb, 2015). Researchers have used SeNPs as carriers of pharmaceutical agents to enhance their bio-efficacy (Liu et al., 2012; Yu et al., 2014). This strategy brings new horizon for biomedical therapies and opens a new window for application of SeNPs. But, the SeNPs exhibit a narrow margin between the pharmacological and toxicological effects. Besides, the QS activity of SeNPs-based anti-QS nano-inhibitors remains unexplored. However, a QS-targeting design through a combination with QS inhibitor could be a good tactic to overcome the detrimental side effects of SeNPs.

A considerable body of historical evidences exist describing the use of honey in wound treatment thus emerging as an antimicrobial since ancient times (Zumla and Lulat, 1989). It has gained new attention in the fight against drug-resistant bacteria by modulating their QS signaling (Truchado et al., 2009; Jadaun et al., 2015). The studies linearly correlated its anti-QS activity with total and individual phenolic compounds. However, the narrow bioavailability of these compounds limits their efficacy in both *in-vitro* and *in-vivo* system. In the present study, we look forward to design the scaffold of SeNPs (carriers) and phytochemicals of honey (HP; anti-QS agents), conjugated on the surface of SeNPs (SeNPs@HP). In which SeNPs could enhance the anti-QS activity of HP against *P. aeruginosa*. *In-vitro* anti-virulence activity and the underlying mechanisms of SeNPs@HP were also examined.

## Results

### Bio-fabrication and characterization of SeNPs@HP

**Figure 1:**
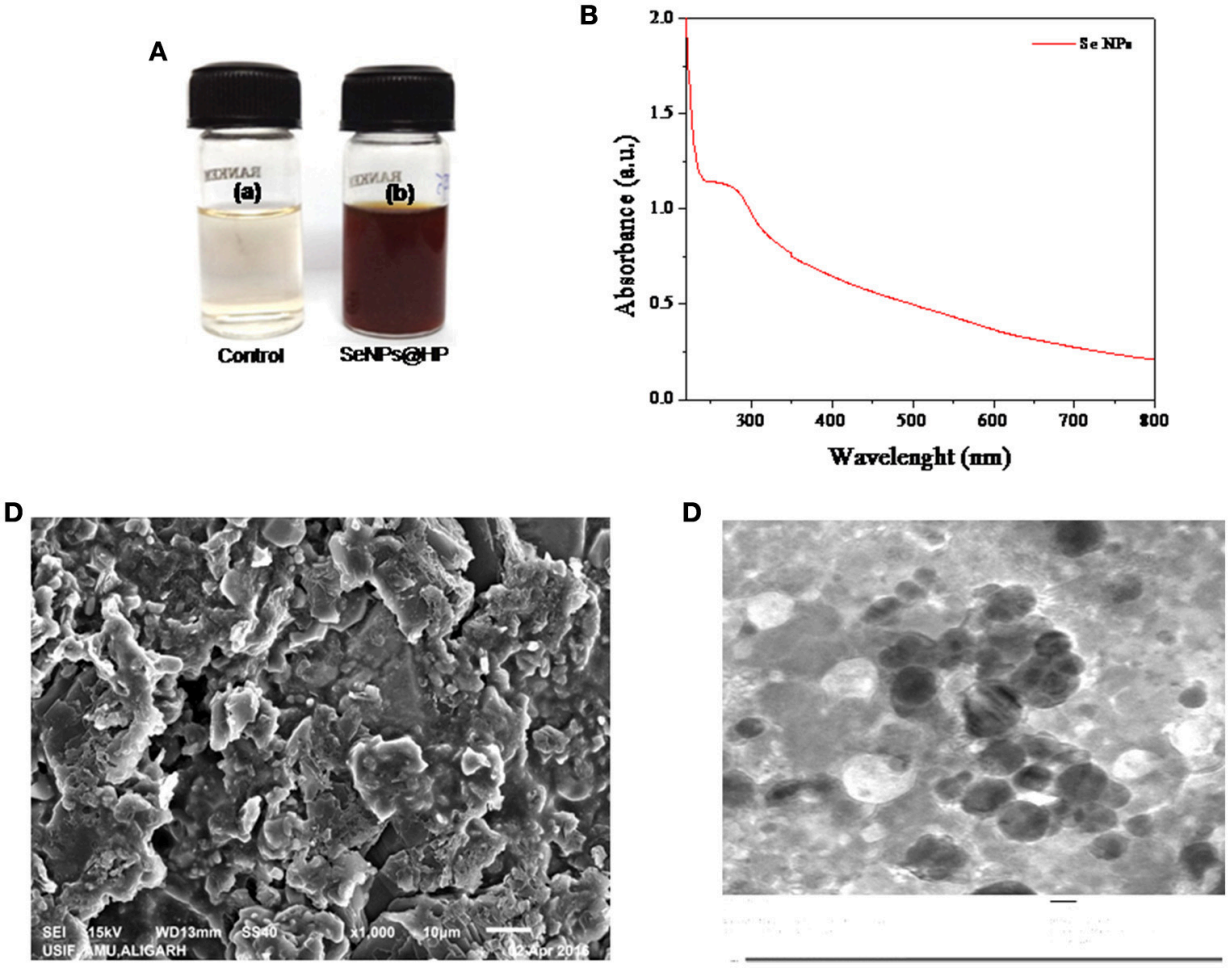
**Biofabrication and characterization of SeNPs@HP. (A)** Photographs of the results for 30 min storage after the redox reaction in the absence **(a)** and presence **(b)** of HP. **(B)** UV-vis spectrum of SeNPs@HP. **(C)** SEM image of SeNPs@HP, which shows the formation of aggregates. **(D)** TEM image of SeNPs@HP. The particles were almost spherical in shape and the particle size ranged from 12 to 15 nm.

Addition of HP solution reduced sodium selenite to form SeNPs. The development of red color in the aqueous medium after an incubation period of 30 min (Figure 1A) suggested the reduction of Se (IV) to Se (0) (Klonowska et al., 2005). Color change might be due to the surface plasma resonance (SPR) with a broad peak (Estevez et al., 2014). A signature peak of absorption maximum at ~265 nm indicated the small particle size of SeNPs (Figure 1B). Similar absorption maximum (λ_max_) has been reported in a study conducted by Fesharaki and colleagues reported at 265 nm (Fesharaki et al., 2010).

Electron microscopy analysis was performed to examine the structure and size of the SeNPs@HP. The Scanning electron microscopy (SEM) images indicated that the NPs were forming aggregates (Figure 1C). After incubation of sodium selenite with HP for 30 min, the sample solution was harvested for the transmission electron microscopy (TEM) analysis. Figure 1D depicts the TEM image of SeNPs@HP. The results showed that the SeNPs existed as well-dispersed spherical particles. By counting ~200 NPs in numerous TEM micrographs, the statistical data revealed the mean particle size of 13.5 nm and standard deviation of 1.10 nm.

**Figure 2:**
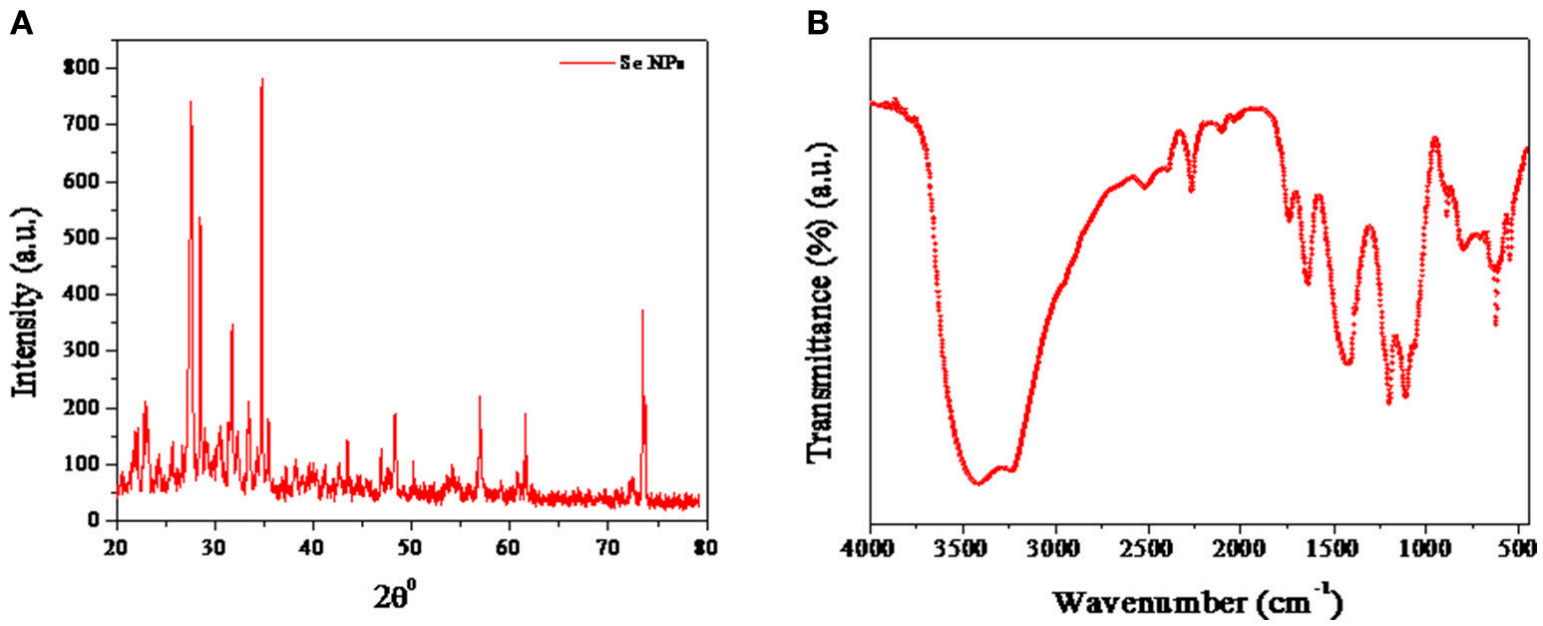
**Characterization of SeNPs@HP. (A)** XRD pattern of SeNPs@HP. **(B)** FTIR analysis of SeNPs@HP showing peaks at 3,420 cm^−1^, corresponding to the O-H stretch; 1375 cm^−1^, the C-H stretch and 1,050 cm^−1^, the C-O stretch.

The crystal structure and phase composition of SeNPs@HP was determined using X-ray diffraction (XRD) technique shown in Figure 2A. The results confirmed the crystalline nature of synthesized nanomaterial. The diffraction peaks at 23.2°, 27.6°, 43.6°, 56.8°, and 62.3° could be indexed to the crystal planes of (100), (101), (111), (200), (2 2 0) crystalline Se which were in good agreement with JCPDS (file no.-06-0362). The estimated average lattice constant was *a* = 4.363 A° which matched very well with the reported JCPDS data. The calculated average particle size of SeNPs@HP was 12.4 nm.

RP-HPLC of SeNPs@HP suggested that there were some PPs associated with the NPs. The releasing of HP from the SeNPs was studied in absolute ethanol solution at 40 ± 1°C and characterized by HPLC after 1 h. The HPLC chromatogram revealed the presence of caffeic acid, quercetin, kaempferol, acacetin, apigenin, chrysin, pinocembrin, and pinobanksin (Supplementary Figure 1). Thus, nano-Se could be used as efficient carriers for the HP. Moreover, Fourier transformed infrared spectroscopy (FTIR) analysis showed that the SeNPs had some functional groups deposited on their surfaces. Most of the peaks represented hydroxyl (OH) groups: the peak at 3,420 cm^−1^ attributed to the OH stretch; the peak at 1,375 cm^−1^ represented the phenolic OH; and the peak at 1,050 cm^−1^represented the C-O stretch (Figure 2B). The other peaks of medium intensity were due to −CH_3_ and OCH_3_ groups related with the biopolymers, present in the honey. The results indicate that the honey acts as reducing and stabilizing agents for SeNPs.

### Determination of minimum inhibitory concentration (MIC)

SeNPs, HP, and SeNPs@HP exhibited strong antimicrobial activity on bacteria tested in the study and the MIC values are presented in Table 1. The MIC values for *Chromobacterium violaceum* CV12472 were 20 μg/mL (SeNPs), 2% (HP), and 10 μg/mL (SeNPs@HP), whereas the MIC values for *P. aeruginosa* PAO1 were 25 μg/mL, 5%, and 15 μg/mL, respectively. To detect the effect of sub-MIC of HP on the growth of CV12472 and *P. aeruginosa* PAO1, viable cell count methods were used. The 0.3 × MIC of test samples did not have any antibacterial activity against *P. aeruginosa* PAO1 and CV12472. These data revealed that the SeNPs, HP, and SeNPs@HP on concentrations lower than 7.5 μg/mL, 0.6%, and 4.5 μg/mL, respectively did not have inhibitory effect on the growth rate of *P. aeruginosa* PAO1 and CV12472 (Table 1).

**Table 1:** **Minimum inhibitory concentration (MIC) and sub-MIC of samples against CV12472 and *P. aeruginosa* PAO1**.

	**CV12472**	***P. aeruginosa* PAO1**
**MIC CONCENTRATION**		
SeNPs (20 μg/mL)	–	
HP (2%)	–	
SeNPs@HP (10 μg/mL)	–	
SeNPs (25 μg/mL)		–
HP (5%)		–
SeNPs@HP (15 μg/mL)		–
**SUB-MIC (NONTOXIC) CONCENTRATION**		
SeNPs (7.5 μg/mL)	+	+
HP (0.6%)	+	+
SeNPs@HP (4.5 μg/mL)	+	+

### Interruption of QS by SeNPs@HP

**Figure 3:**
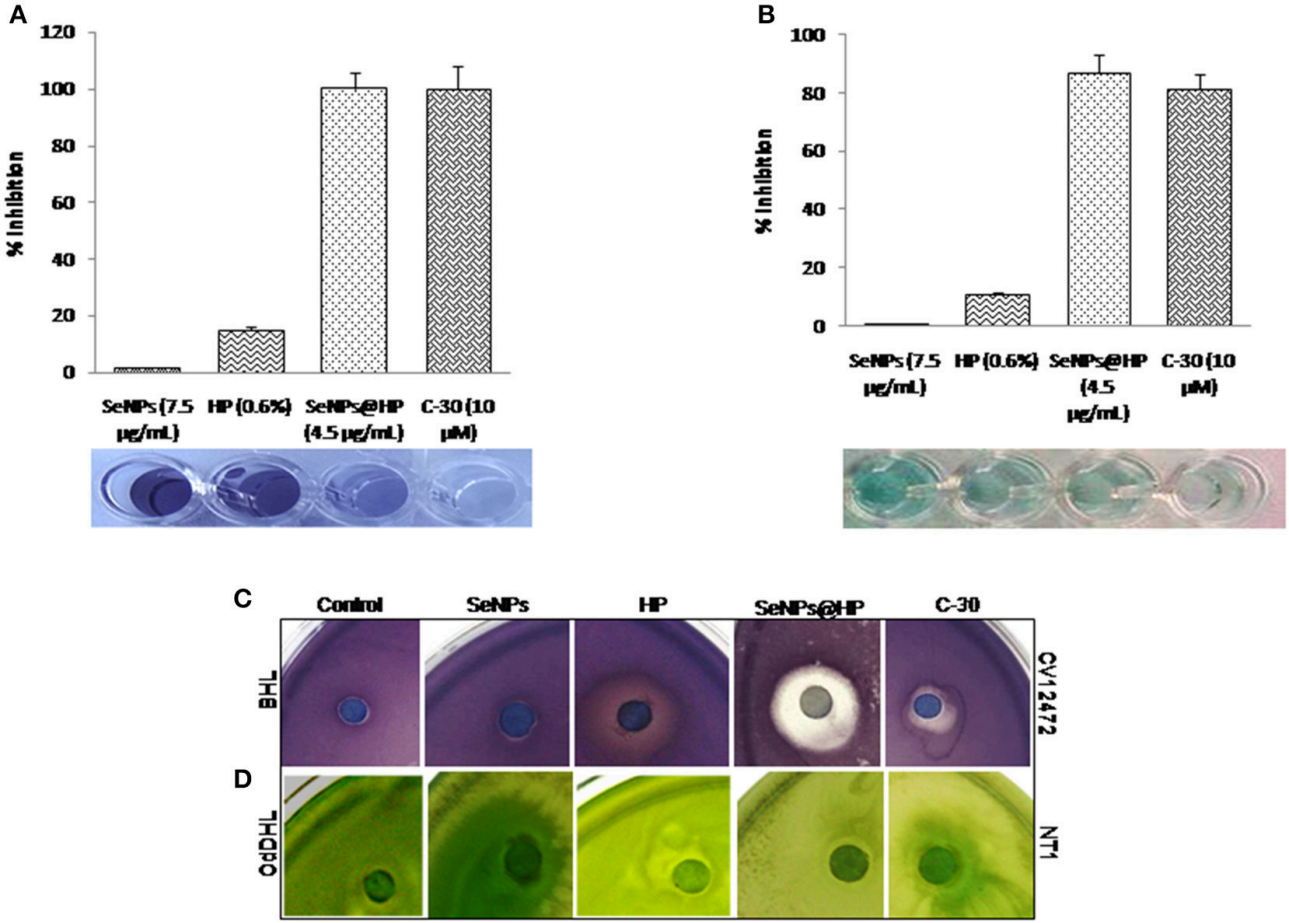
**Inhibition of QS by SeNPs@HP**. Bioindicator strains of bacteria, *C. violaceum* CV12472 and *A. tumefaciens* NT1 were used for assaying anti-QS activity. Inhibition of color changes due to treatment of SeNPs (7.5 μg/mL), HP (0.6%), SeNPs@HP (4.5 μg/mL), and furanone C-30 (10 μM) was quantified by measuring OD at 590 nm for CV12472 and 545 nm for NT1 bacteria. **(A)** Test samples-BHL competition and **(B)** test samples-OdDHL competition. Error bars indicate the standard deviations of 8 measurements. Inhibition of QS potential based on color inhibition was further examined on solid culture media and detail protocol has been described in Materials and Methods section. **(C)** Test samples-BHL competition against CV12472 and **(D)** test samples-OdDHL competition against NT1.

The potency of QS interruption by the SeNPs@HP was firstly explored using an AHL-based *in-vitro* QS competition assay against three genetically modified strains, *C*. *violaceum* (CV) 26 and *Agrobacterium tumefaciens* NT1. In the current investigation, CV26 was used to test QS inhibition associated with *N*-butanoyl-L-homoserine lactone (BHL) production, whereas, *A. tumefaciens* NT1 was used to test QS inhibition associated with *N*-(3-oxododecanoyl)-l-homoserine lactone (OdDHL). TheCV026 strain is a mutant which produces the AHL receptor CviR, that could sense exogenous AHLs having a shorter carbon chain length including BHL. The *A. tumefaciens* NT1 strain produces the AHL receptor TraR, for the detection of AHLs including OdDHL. Although these strains do not produce the exact LasR and RhlR receptors found in *P. aeruginosa*, the TraR and CviR receptors are considered adequate indicators of LasR and RhlR behavior, respectively. The assay was based on comparing the competitive binding of the AHL molecules of *P*. *aeruginosa* (e.g., BHL and OdDHL) and HP to the AHL receptors in which the competition was quantified using UV-vis spectrophotometer (Figure 3A) as well as by measuring zone of inhibition on solid media (Figure 3B). Furanone C-30 was used as the positive control for experiment. In this assay, the development of color indicates the binding of the AHLs to their receptor (AHL), whereas the absence of color represents the lack of AHL and its receptor binding. As shown in Figure 3C, the CV12472 culture had decreased purple pigment, violacein, when was treated with SeNPs@HP. Similar results were observed for the QS inhibition assays using NT1 in which cyan color was diminished by increasing SeNPs@HP levels (Figure 3C). These results demonstrated that SeNPs@HP could interfere with the interaction between the added AHLs (e.g., BHL and OdDHL) and their receptors (e.g., CviR for CV12472 and TraR for NT1).

### Inhibition of biofilm formation by SeNPs@HP

**Figure 4:**
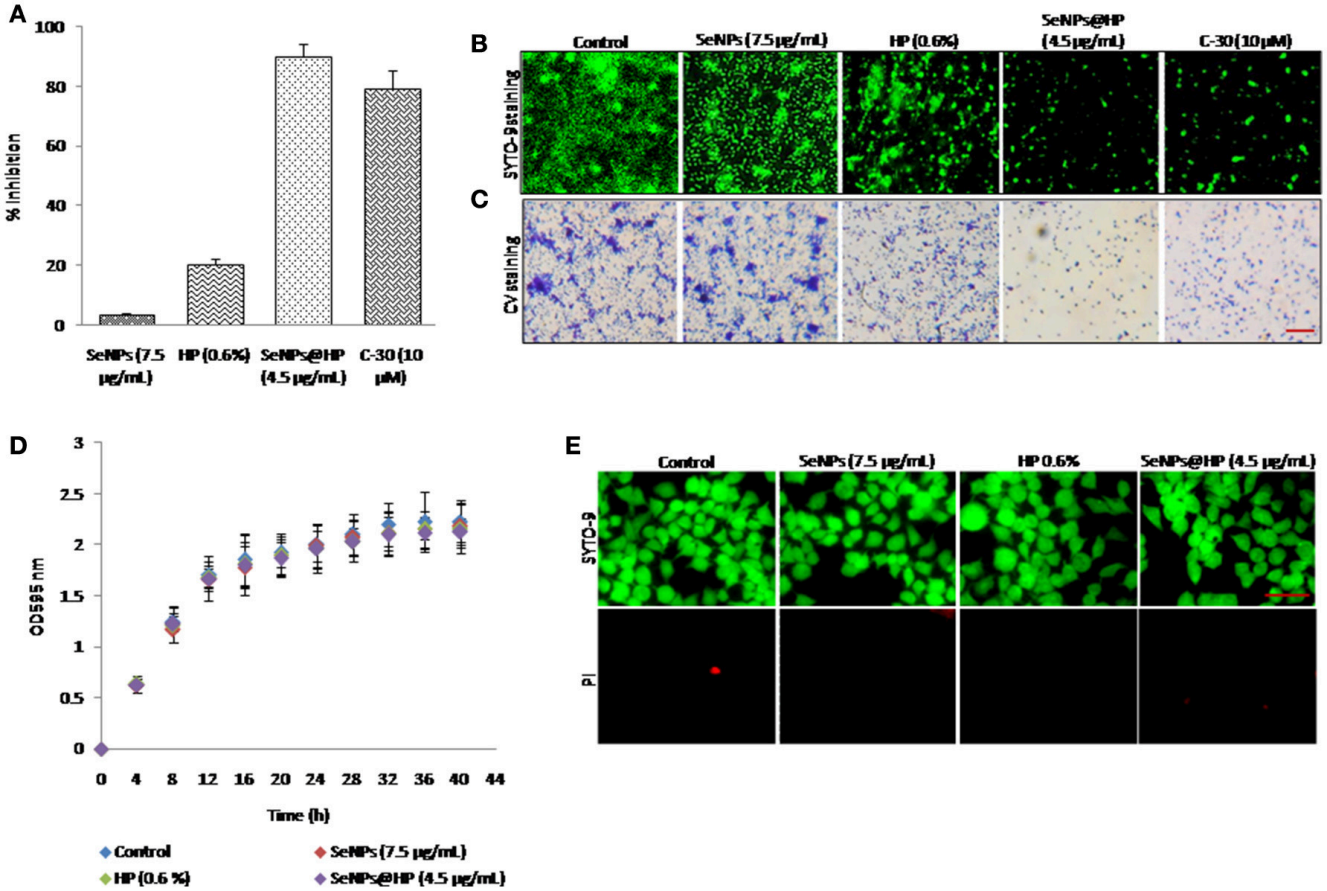
**Anti-biofilm potential of SeNPs@HP. (A)** Inhibitory action of SeNPs (7.5 μg/mL), HP (0.6%), SeNPs@HP (4.5 μg/mL), and furanone C-30 (10 μM) on *P. aeruginosa* PAO1 biofilm formation was determined using CV staining assay for 24 h in MTP. Error bars indicate the standard deviations of 8 measurements. **(B)** Fluorescent and **(C)** light microscopic images of *P. aeruginosa* PAO1 biofilms developed on cover glass slides in the presence of SeNPs (7.5 μg/mL), HP (0.6%), SeNPs@HP (4.5 μg/mL), and furanone C-30 (10 μM) and DW treated control. The biofilm cells were stained with SYTO-9 and CV and analyzed observed green fluorescence and phase contrast lights, respectively. The DW was used to maintain untreated control. The scale bar: 5 μm. **(D)** Growth curve analysis of *P. aeruginosa* PAO1 in the presence of SeNPs (7.5 μg/mL), HP (0.6%) and SeNPs@HP (4.5 μg/mL) for 40 h in flask. Error bars indicate the standard deviations of three measurements. **(E)** Cytotoxic effect of SeNPs (7.5 μg/mL), HP (0.6%) and SeNPs@HP (4.5 μg/mL) on human lung epithelial cells for 24 h. The cells growing on cover slips were stained with SYTO-9 green for live cells and with PI red for dead cells and analyzed under a fluorescence microscope at 20x magnification. The scale bar: 50 μm.

Biofilm formation is regulated by QS signaling in bacteria. Therefore, the effect of SeNPs@HP on biofilm formation in *P. aeruginosa* PAO1 was assessed after 24 h of incubation. Biofilm formation was reduced >90% by the treatment of 4.5 μg/mL SeNPs@HP as shown in Figure 4A. The inhibition of biofilm formation by SeNPs@HP was also demonstrated on glass cover slides microscopically. Figure 4B shows confocal laser scanning microscope (CLSM) images of *P. aeruginosa* PAO1 biofilms formed. The biofilms treated with 4.5 μg/mL of SeNPs@HP was observed to be shallower and less dense than the control biofilm. Crystal violate (CV) staining assay was further confirmed the potent inhibitory effect of SeNPs@HP on *P. aeruginosa* biofilm formation (Figure 4C). However, SeNPs and HP alone were unable to show promising anti-biofilm potential. Next, the effects of SeNPs, HP, and SeNPs@HP on *P. aeruginosa* PAO1 growth were also assessed by recording the absorbance of batch cultures at A595 nm (Figure 4D). The growth of bacterium was not significantly different between the treated and untreated batches during the lag and exponential growth phases. In addition, live and death cell staining assays were performed using SYTO-9 and propidium iodide, respectively on human lung epithelial cells. No cytotoxicity was observed in SeNPs and SeNPs@HP-treated cells (Figure 4E), suggesting non-toxic effect of both nanomaterials.

### Inhibition of QS-regulated virulence by SeNPs@HP

**Figure 5:**
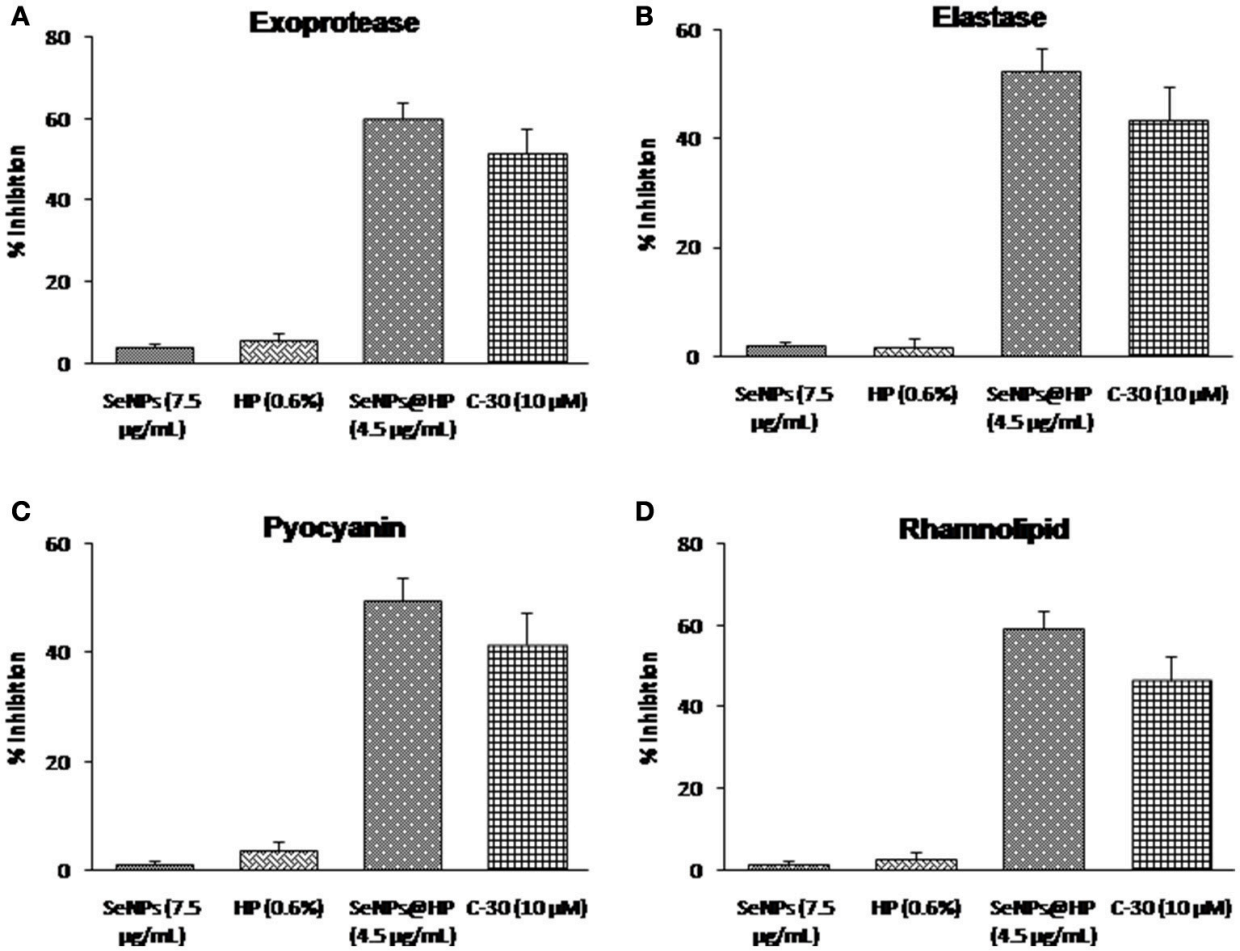
**Impact of SeNPs@HP on the secretion of virulence factors**. Levels of virulence factors such as **(A)** exoprotease, **(B)** elastase, **(C)** pyocyanin, and **(D)** rhamnolipid were measured in SeNPs (7.5 μg/mL), HP (0.6%), SeNPs@HP (4.5 μg/mL), and furanone C-30 (10 μM) treated or untreated culture filtrates of *P. aeruginosa* PAO1. Error bars indicate the standard deviations of 8 measurements.

QS-dependent production of virulence factors namely exoprotease, elastin-degrading elastase, pyocyanin, and rhamnolipid were analyzed to assess the effects of SeNPs@HP on virulence. As shown in Figure 5, SeNPs@HP clearly reduced the production of virulence factors in the supernatant of SeNPs@HP-treated *P. aeruginosa* PAO1. At 4.5 μg/mL of SeNPs@HP, 60.2% inhibition for protease activity, 52.7% reduction for elastase activity, 49.6% inhibition for pyocyanin content, and 59.6% suppression for rhamnolipid production were observed (Figures 5A–D). However, secretion of virulence factors in SeNPs and HP alone treated *P. aeruginosa* PAO1 was not significantly different from untreated control. The significant inhibition of swarming motility in *P. aeruginosa* PAO1 was also observed, when exposed to 4.5 μg/mL of SeNPs@HP. In the presence of SeNPs@HP, the bacterium was unable to perform swarming motility on LB agar plates at the point of inoculation in the center with a diameter not exceeding 5 mm (Supplementary Figure 2), and tendril formation or other features indicating of swarming motility were not observed.

### SeNPs@HP induces wound healing and prevents *P. aeruginosa* PAO1 killing of mouse

**Figure 6:**
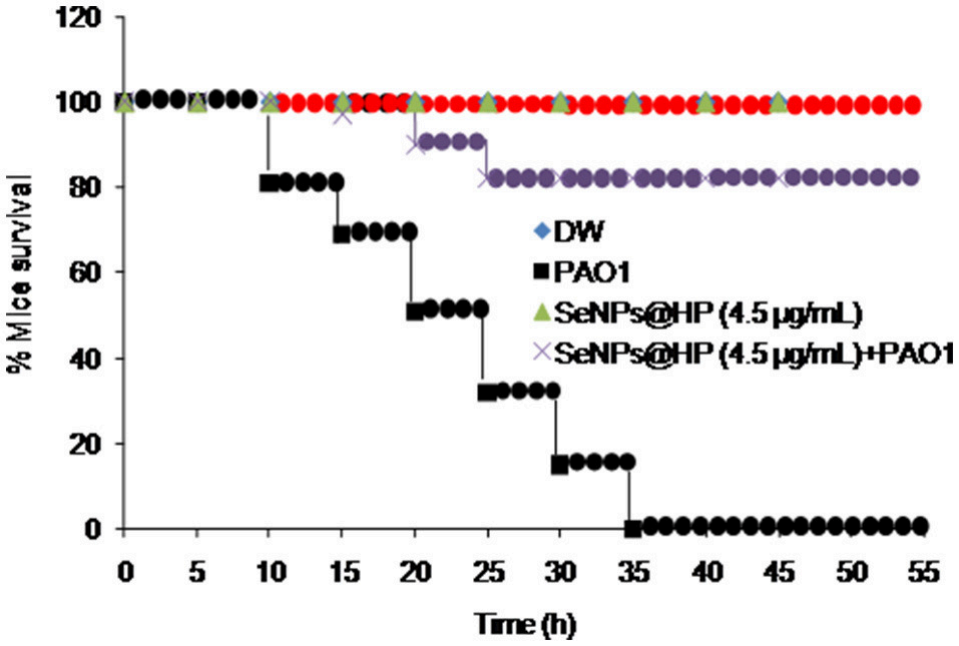
**Determination of *in-vivo* anti-QS activity of SeNPs@HP using mice mortality assay**. Cell suspension (2.0 × 10^7^ cfu/mL) was prepared from overnight grown culture of *P. aeruginosa* and injected into the abdominal cavity of 4 weeks old pathogen free female mice. Mortality was measured during a 45 h period for 12 mice for each treatment. DW and SeNPs@HP in DW was injected into mice as negative controls.

Next, to determine whether our designed SeNPs@HP could reduce pathogenicity of *P. aeruginosa* PAO1, we used mouse infection assay. Skin wounds were made on the top side of mouse and bacterial infection was initiated by applying 1 × 10^7^ colony forming unit (cfu)/mL of *P. aeruginosa* PAO1. Then wounds were treated with various concentrations of the SeNPs@HP. Sterilized distilled water (DW)-treated mice were used as untreated controls. After 2, 4, and 6 days of infection, the cfu/mL of the bacterium from wounds was determined. There was no statistically significant difference between all groups on the first day post infection, however, the bacterial number in wound area of SeNPs@HP-treated group decreased significantly on day 4 and 6 post-infection as compared to DW-treated control group (Table 2). In the process of wound healing, bacterial infection to skin wounds leads to a significant delay in the closure of excisional wound sites as compared with non-infected wound sites. When SeNPs@HP was used to treat the infected wound skin sites, wound healing process was dramatically enhanced. The area of wound was measured after 5, 10, 15, and 20 days of post infection in all groups. A significant rate of closure of wound was observed between 15 and 20 days post-surgery (*P* < 0.01). The percentage of wound healing of SeNPs@HP against the post infection days was presented in Table 3. Mice infected with *P. aeruginosa* PAO1 only started to die after 10 h of incubation and 100% mortality was recorded after 35 h. Although SeNPs@HP-treated mice showed 82% survival rate, but SeNPs and HP groups did not show significant protection from killing effect of *P. aeruginosa* PAO1 (Table 3). Mice treated with SeNPs@HP were alive at the end of the incubation of 55 h (Figure 6).

**Table 2:** **Bacterial load of wound area post *P. aeruginosa* PAO1 infection**.

**Treatment**	**Cfu/mL (Log_10_)**		
	**2nd day**	**4th day**	**6th day**
DW+PAO1 infected	5.9 ± 0.41	7.3 ± 0.62	8.1 ± 0.47
SeNPs (7.5 μg/mL) + PAO1 infected	6.0 ± 0.22	7.1 ± 0.85	7.9 ± 0.82
HP (0.6%) + PAO1 infected	5.8 ± 0.52	6.3 ± 0.27	6.2 ± 0.58^*^
SeNPs@HP (4.5 μg/mL) + PAO1 infected	5.3 ± 0.21	5.1 ± 0.15	4.3 ± 0.26^**^

*Results are expressed as mean ± SEM. (n = 6)*.

* P < 0.05;

** *P < 0.01 when compared to control group (one-way ANOVA followed by t-test)*.

**Table 3:** **Effect of SeNPs@HP on the wound healing in wound dripped with *P. aeruginosa* PAO1**.

**Treatment**	**Wound healing (%)**			
	**5th day**	**10th day**	**15th day**	**20th day**
DW+PAO1 infected	14.4 ± 0.92	41.7 ± 1.93	57.5 ± 2.73	75.5 ± 4.72
SeNPs (7.5 μg/mL)+ PAO1 infected	13.6 ± 0.63	45.6 ± 2.52	64.7 ± 3.72	78.3 ± 4.72
HP (0.6%)+ PAO1 infected	15.0 ± 1.03	48.7 ± 3.75	69.6 ± 5.71	81.2 ± 6.12
SeNPs@HP (4.5 μg/mL) + PAO1 infected	31.7 ± 0.63^*^	69.6 ± 2.59^*^	81.6 ± 6.24^**^	97.3 ± 3.16^**^

*Results are expressed as mean ± SEM. (n = 6)*.

* P < 0.05;

** *P < 0.01 when compared to control group (DW+PAO1 infected) (one-way ANOVA followed by t-test)*.

### Down-regulation of QS-related virulence genes by SeNPs@HP

**Figure 7:**
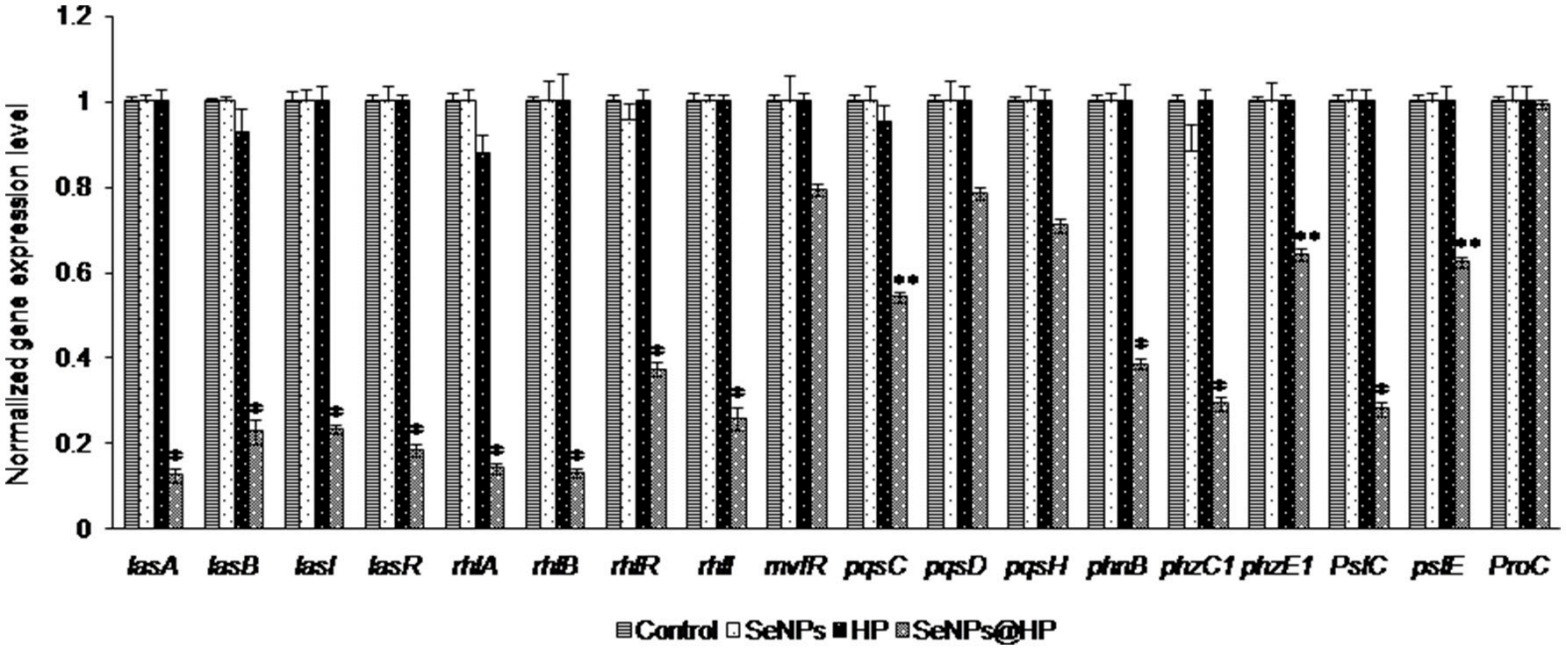
**Inhibitory effects of SeNPs@HP on the expression of QS-inducible genes through RT-qPCR analysis**. RNA was extracted and employed for synthesizing cDNA from treated and untreated biofilm cells with SeNPs (7.5 μg/mL), HP (0.6%), and SeNPs@HP (4.5 μg/mL) for 24 h. The relative magnitude of gene expression level was defined as the copy number of cDNA of each gene in the biofilm cells normalized by the copy number of cDNA of the corresponding gene in biofilm cells without test sample. Error bars indicate the standard deviations of 3 measurements. ^**^p < 0.001 vs. the control. ^*^p < 0.01 vs. the control.

*P. aeruginosa* PAO1 biofilmed cells were analyzed by RT-PCR technique to identify the genes targeted by SeNPs@HP and to explore the molecular mechanism that decreased biofilm formation and virulence when SeNPs-HP was supplemented. As shown in Figure 7, QS-regulated genes of *P. aeruginosa* PAO1 were reduced on treatment with SeNPs@HP. The genes involved in the production of virulence factors such as rhamnolipid, elastase, and pyocyanin were highly repressed. Many QS-inducible genes such as *lasA, lasB, lasI, lasR, rhlA, rhlB, rhlR, rhlI, mvfR, pqsC, pqsD, phnB, pqsH, phzC1, phzE1, pslC*, and *pslE* were targeted, and the expression pattern of these genes were determined in *P*. *aeruginosa* biofilm cells treated with SeNPs, HP, and SeNPs@HP. All of the targeted QS-regulated genes were significantly down expressed in the biofilm cells with SeNPs@HP ranged from 21 to 87% compared with DW-treated and untreated control. The expression of *lasA, lasB, lasI, lasR, rhlA, rhlB, rhlR, rhlI, phzB, phzC1*, and *pslC* genes were comparatively more down-regulated (61–87%) than *mvfR, pqsC, pqsD, phnB, phzE1 pqsH*, and *pslE* genes (21–46%). The expression of the *proC* housekeeping gene was not strongly affected by SeNPs@HP. Interestingly, SeNPs and HP alone were unable to alter the expressions of above genes.

### Computational molecular docking analysis

**Figure 8:**
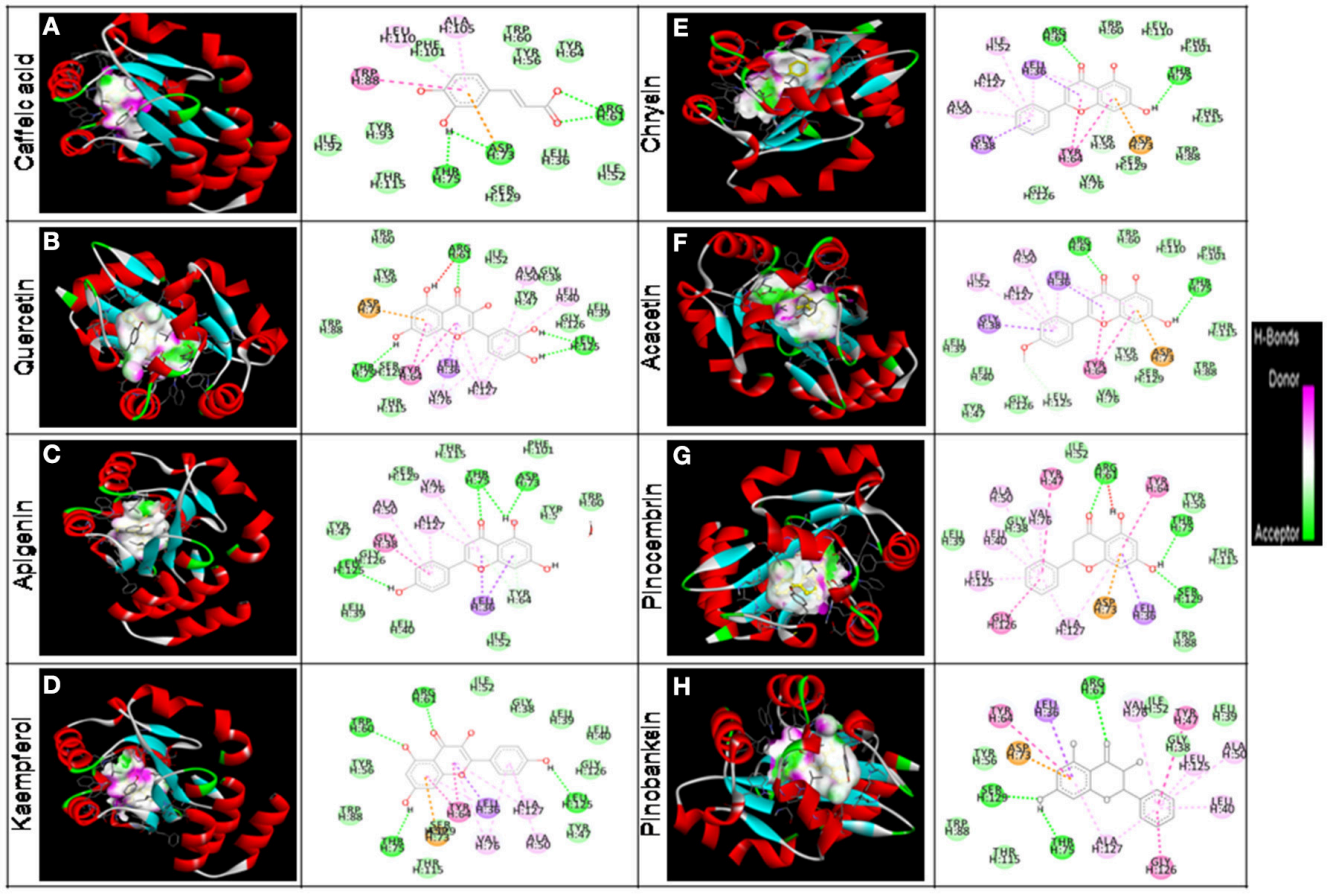
*****In silico*** molecular docking analyses for determination of interaction between HP and LasR protein**. HP are bounded (black color) in the binding cavity of LasR protein and colored by hydrogen bond character, with receptor donor colored in pink and receptor acceptor in green. A 3D depiction of binding orientation in LasR protein by **(A)** caffeic acid, **(B)** quercetin, **(C)** apigenin, **(D)** kaempferol, **(E)** chrysin, **(F)** acacetin, **(G)** pinocembrinand **(H)** pinobanksin. Hydrogen bondings and hydrophobic interactions between the surrounding amino acid residues and the ligand are also displayed.

In RT-PCR-based gene expression analysis, we found down-regulation of QS-related gene expression in bacterial biofilm cells. To investigate the possibility of binding interference between OdDHL and its cognate signal receptor, LasR, by polyphenols of honey, we performed detailed computational molecular docking studies. A PDB coordinate file of LasR (PDB ID: 2UVO) was downloaded and employed for the docking studies. 2UVO is the X-ray structure of *N*-terminal OdDHL binding domains of LasR in complex with OdDHL. We used the AutoDock 4.2 docking tools to see the interaction between the receptor (LasR) and its ligand (OdDHL). Due to the reproducibility of the AutoDock module to correctly dock the crystal ligand for the LasR receptor, docking studies of HP were performed with the same default settings. Docking of HP including caffeic acid, quercetin, kaempferol, acacetin, apigenin, chrysin, pinocembrin, and pinobanksin in the OdDHL binding site of the LasR protein indicated that all polyphenols made hydrogen bonding and hydrophobic interactions (Figure 8). The best-docked acacetin fit inside the OdDHL binding site of the LasR protein. Acacetin made hydrogen-bonding interactions with the Trp60, Arg61, and Thr75 and hydrophobic interactions with Leu36, Tyr64, Ala50, Ile52, and Ala127. Trp60 and Arg61 were key amino acids to make hydrogen-bonding interactions with the X-ray crystal ligand OdDHL. The calculated *E*_binding_ value of acacetin was found to be −9.41 kcal/mol. Based on *E*_binding_ values of phytochemicals, the order was found to be apigenin (−9.20 kcal/mol) >pinocembrin (−8.90 kcal/mol) >chrysin (−8.83 kcal/mol) >pinobanksin (−8.51 kcal/mol) >quercetin (−8.21 kcal/mol) >caffeic acid (−6.40 kcal/mol) >kaempferol (−5.97 kcal/mol) (Table 4). The HP interaction with the OdDHL binding site of LasR might be the reason for the inhibition of virulence of *P. aeruginosa* PAO1.

**Table 4:** ***E*_binding_ values of HP**.

**Phytochemical**	**Binding energy (kcal/mol)**	**Inhibition constant (nm)**
Acacetin	−9.41	127.17
Apigenin	−9.20	180.42
Pinocembrin	−8.90	299.38
Chrysin	−8.83	336.92
Pinobankis	−8.51	577.58
Quercetin	−8.21	936.39
Caffeic acid	−6.40	20.42
Kaempferol	−5.97	42.09

## Discussion

*P. aeruginosa* is recognized as one of the most key pulmonary bacterial pathogens and the major cause of morbidity and mortality in cystic fibrosis. It can colonize on lung surfaces by forming a biofilm in which bacterial cells attach together and are fixed within a self-developed extracellular matrix. Biofilm cells of *P. aeruginosa* are reported to be several times more resistant to antibiotics than planktonic cells, which often cause difficulties in destroying them from infected patients. *P. aeruginosa* uses QS systems for the production of virulence factors and biofilm formation (Shirtliff et al., 2002; Gonzalez and Keshavan, 2006). Interruption of QS in *P. aeruginosa* is considered as a new approach for the development of potential anti-pathogenic therapy. In the current investigation, we have demonstrated that SeNPs@HP treatment of the clinically isolated strain PAO1 of *P. aeruginosa* could inhibit biofilm formation and virulence *in-vitro*. The bacterium exposed to SeNPs@HP also exhibited reduced virulence in mice infection model.

The SeNPs act as efficient carriers for HP such as caffeic acid, quercetin, kaempferol, acacetin, apigenin, chrysin, pinocembrin, and pinobanksin which identified by HPLC analysis. HP have significant antimicrobial uses against different drug resistant pathogenic bacteria, particularly anti-QS action (Israili, 2014; Khan et al., 2014). But, the poor water solubility, limited bioavailability, and poor stability of phytomolecules usually hinder their full anti-QS potential (Munin and Edwards-Levy, 2011). The development of nano-sized delivery system represents a significant progress of the scientific approach to enhance the biological properties of phytochemicals (Munin and Edwards-Levy, 2011; Shakeri and Sahebkar, 2015). This study represents first report on selenium-based nano drug delivery system for HP and clearly demonstrates that our designed SeNPs@HP has the ability to inhibit the QS system of wild type strain of *P. aeruginosa* PAO1 more efficiently, as compared to SeNPs and honey alone. This might be due to efficient delivery as well as enhanced bioavailability of HP. This result corroborates well with the finding of others. Nano drug delivery approaches confer a wide range of advantages, such as assisted transport of incorporated phytomolecules across the cell membrane, enhanced penetration potential into biofilm of bacteria and enhanced bioavailability, targeted drug delivery, protection of sensitive drug agents from biological and environmental degradation, and sustainable release of anti-virulence agent (de la Zerda and Gambhir, 2007; Fernandes et al., 2010; Zhu et al., 2013).

Inhibition of QS system can be attained either through interruption of AHLs synthesis, or interruption of AHLs to conjugate with the signal receptor (Kim et al., 2015; Singh B. R. et al., 2015). Our results indicated that the anti-QS property of HP on *P. aeruginosa* began with binding to the receptor LasR, which was evidenced by the molecular docking analysis. *In-silico* docking studies of HP such as caffeic acid, quercetin, apigenin, kaempferol, chrysin, acacetin, pinocembrin, and pinokanksin with LasR showed the potential binding mode of HP to the active site of the ligand-binding domain. The conjugating domain of HP was analogous to that of the crystal ligand OdDHL, through a combination of hydrogen bonds and hydrophobic interactions. Recent wide-ranging structure-activity relationship investigations of OdDHL resemblances revealed that the replacement of the homoserine lactone moiety (present in OdDHL) with a substituted aromatic system could alter the QS agonistic properties into antagonistic properties (Kim et al., 2015). Thus, the substituted phenyl ring of HP might contribute to its antagonistic properties. In particular, the phenolic hydroxyl group of HP that made direct hydrogen bonding interactions with amino acids, was observed in the crystal LasR and OdDHL complex. Moreover, attenuation of QS in *C. violaceum* and *A. tumefaciens* was not due to the reduction of AHLs production and by degrading AHLs, but obstruction of AHLs receptors by HP. To obtain direct evidence that LasR is the target of HP, we also evaluated SeNPs@HP inhibition of biofilm formation in *Escherichia coli* transformed by a plasmid overexpressing LasR. The transformed *E. coli* was still exhibited biofilm formation, when treated with the SeNPs@HP, suggesting that the HP targeted LasR in *E. coli* (Supplementary Figure 3). Moreover, effects of SeNPs@HP on the production of virulence factors and anti-biofilm activity were further assessed in *lasR* null mutant strain of *P*. *aeruginosa* PAO1. The results demonstrated that the SeNPs@HP treatment induced inhibition of VFs (elastase, protease, pyocyanin, and rhamanolipid) and biofilm formation, but these effects of SeNPs@HP can be significantly reduced in LasR mutant (Supplementary Figures 4, 5). The data concluded that SeNPs@HP inhibit *P*. *aeruginosa* QS signaling via LasR.

The binding of SeNPs@HP to the LasR signal receptor causes it to lose its function as a transcriptional activator. The down expression of *lasA, lasB, lasI, lasR, rhlA, rhlB, rhlR, rhlI, mvfR, pqsC, pqsD, phnB, pqsH, phzC1*, and *phzE1* in the RT-qPCR result supports our assumption. *P. aeruginosa* secretes toxic compounds and degradative enzymes such as elastase, LasA protease, and pyocyanin that also contribute to pathogenesis (Christensen et al., 2013; O'Loughlin et al., 2013). We found that the SeNPs@HP could reduce the secretion of total protease, elastase, pyocyanin, biofilm formation, and swarming motility in *P. aeruginosa* PAO1 without inhibiting its growth. Down expression of these genes might interfere with the normal secretion of several virulence factors including exoprotease, rhamnolipid, pyocyanin, and swarming motility via the actions of *lasAB, rhlAB, phzA1-G1* operons, respectively. Reduced secretion of these virulence factors by SeNPs@HP supported our speculation. The expression of rhlR triggers the production of BHL signal receptor, RhlR. BHL and RhlR complexes are responsible for the transcriptional activation of *rhlAB* operon and the transcriptional repressor of *mvfR* gene. Hence, binding of HP to the LasR signal receptor could cause a decrease in RhlR production that could result in a decrease in the *rhlAB* operon and *rhlI* production while eliminating transcriptional repression of *mvfR*. Related to earlier investigations, cationic Se (Dutta and Willcox, 2014), quercetin (Singh B. N. et al., 2015), kaempferol (Vikram et al., 2010), apigenin (Nazzaro et al., 2013), and caffeic acid (Borges et al., 2014) exhibited significant anti-QS effects. They can significantly reduce the secretion of virulence factors and biofilm formation in human pathogenic bacteria. All these findings strongly suggest that polyphenols anti-pathogenic effect is due to QS interruption.

Nevertheless, since all the results obtained from *in-vitro* studies always cannot be reproduced under *in-vivo* conditions. In this context, we used excision wound rat–*P. aeruginosa* infection model to investigate the anti-pathogenic ability of SeNPs@HP. During the wound healing process, bacterial infection to skin wounds lead to a significant delay in the closure of excisional wound sites, therefore anti-pathogenic tactic can play an important role in wound healing. In this study, we noticed that the cfu/mL of *P. aeruginosa* PAO1 was significantly reduced in wound area on 10th and 15th day of infection in SeNPs@HP-treated groups when compared with the DW-treated control group. Moreover, SeNP@HP was found to reduce the pathogenicity of *P. aeruginosa* PAO1 under *in-vivo* model system, confirmed by mice mortality test. Our results were similar to Yin et al. their study revealed that topical application of tea polyphenols could decrease the systemic spread of *P. aeruginosa* Pa1 in excision wounded infection mice model as depicted by lower bacterial counts in wound area (Yin et al., 2016). This might be the result of QS interruption that led to inhibition of secreted virulence factors, which finally delayed its infection. Wound size was reduced dramatically when exposed to SeNPs@HP as shown in Table 1. Polyphenolic compounds of honey significantly enhanced the quality of wound healing and formation of scar in an incisional wound healing model in rats (Kapoor et al., 2004). Other numerous plant polyphenols also have promising effects on wound contraction and healing (Gomathi et al., 2003; Mandal and Mandal, 2011; Muhammad et al., 2013; Suntar et al., 2013). The present study clearly indicates that HP in nano form at sub-MIC can stimulate wound healing, along with other information about green tea in the literature, strongly suggests that honey polyphenols may be beneficial in wound healing due to their antioxidant, antiseptic, and anti-inflammatory properties and may contribute in recovery of burn wounds and scars (Mandal and Mandal, 2011; Quideau et al., 2011; Singh et al., 2014).

**Figure 9:**
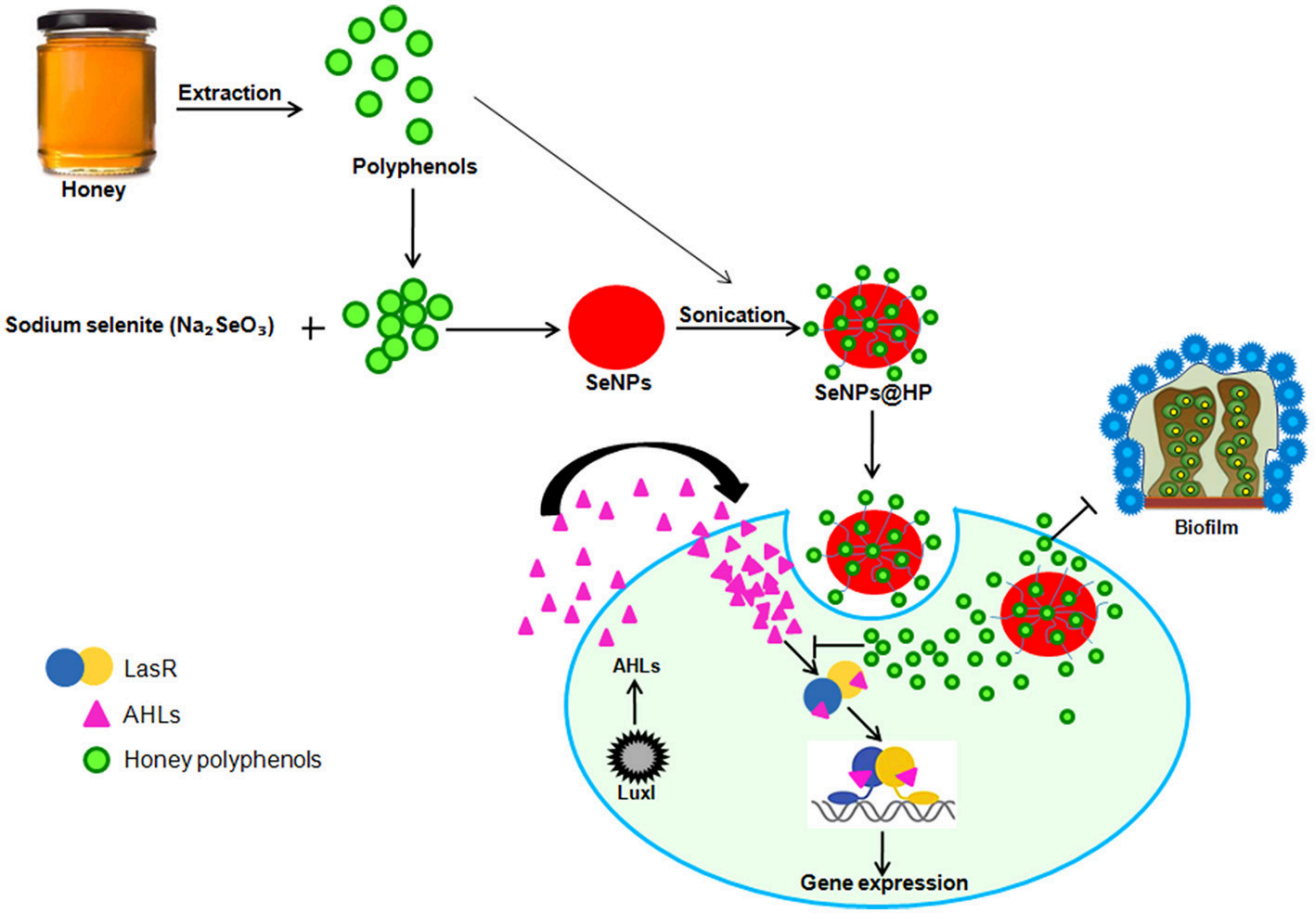
**Pictorial representation for the biofabrication of scaffold of SeNPs and HP (SeNPs@HP) and its anti-QS activity in *P*.* aeruginosa* PAO1**.

The significance of this study is the finding that SeNPs@HP can inhibit the production of QS-regulated virulence factors and biofilm formation via LasR (Figure 9). The SeNPs@HP also helps to reduce the virulence of *P. aeruginosa in-vivo*, which resulted in reduced pathogenicity of this pathogen in mice infection model. Also, this investigation delivers insight into the molecular mechanism of the QS inhibitory effects of SeNPs@HP in *P. aeruginosa* via competitive binding to cognate receptors. These observations suggest that nano-Se could be used as promising carriers to deliver phytochemicals of honey for combating *P. aeruginosa* infections in airway.

## Materials and methods

### Chemicals, strains and culture conditions

Honey of Indian honey bee (*Apis cerana indica*) was obtained from Research Station (Banthra) of CSIR-National Botanical Research Institute, Lucknow, India and furanone C-30, a standard anti-QS compound, was purchased from Sigma Aldrich (St. Louis, MO, USA). *C. violaceum* ATCC12472 and *A. tumefaciens* NT1 strains were generously provided by Prof. Kalai Mathee, USA. Moreover, *P. aeruginosa* PAO1 and LasR over expressing strain of *E. coli* (pSW196.*lasR*) were obtained from Prof. E.P. Greenberg and M. Schuster, USA respectively. Each strain was maintained on a Luria-Bertani (LB) slant at 37°C and for any experiment an active growth for 24 h at 37°C was used.

### Development of nanoscaffold

Honey was used as a rich source of redox active plant polyphenols (PPs) to synthesize SeNPs. For extraction of PPs, 10% solution of honey was prepared in 100 mL distilled water (DW) and mixed vigorously at room temperature (25 ± 1°C). The obtained solution was transferred into the separating funnel and subjected to extraction with the help of two volume (v/v) ethyl acetate. The extraction solution was mixed vigorously for 10 min and separating funnel was then kept undisturbed at 4°C for overnight. The upper phase containing PPs was collected from the separating funnel and dried on rota evaporator (BUCHI, USA) at 30°C. The obtained PPs extract (PPE) was redissolved in 50% hydroalcohalic solvent and used to develop scaffold of SeNPs. For the development of SeNPs with PPE scaffold, obtained PPE extract (5 mg/mL; 10 mL) was added into the aqueous solution of sodium selenite (90 mL) to maintain the 1 mM final concentration. The reaction mixture solution was kept for 24 h at 37°C, after that appearance of red color in the culture flasks suggested the PPE-mediated reduction of Se(VI) to Se(0) or SeNPs and its successful *In-situ* fabrication of PPE scaffold (denoted as “SeNPs@HP”). The developed SeNPs@HP were harvested through the centrifugation at 12,000 g for 12 min and dried under vacuum at 40°C. The obtained dried powder was stored in amber color vial until further use.

### Characterization of nanomaterials

SeNPs@HP were characterized for size, shape, and purity by using various spectrophotometric (UV spectrum, energy ban gap spectroscopy, XRD, FTIR) and microscopic (SEM, TEM) techniques. Synthesis of SeNPs was monitored by measuring UV absorption spectrum analysis (Perkin Elmer CT, USA) in the range 200 to 800 nm wavelength. The samples for TEM were prepared by dispersing the power particles on carbon-coated copper grids. The micrographs were obtained at 200 kV on a JEOL, Tokyo TEM microscope. Images of SeNPs@HP were obtained on a JEOL-JSM-6510LV SEM microscope (Tokyo, Japan) and operated at an accelerating voltage of 20 kV. FTIR (PerkinElmer, CT, USA) analysis in the range of 500 cm^−1^ to 4,000 cm^−1^ using the KBr-disc method was performed to determine the accumulation of HP on the SeNPs by identifying functional groups.

### MIC assay

The MIC of test samples against the selected bacterial strains was tested using a micro-dilution method of Clinical and Laboratory Standards Institute, USA (Clinical and Laboratory Standards Institute, 2009). Briefly, test bacteria having 1–5 × 10^5^ cfu/mL were seeded in 100 μL of LB broth media supplemented with twofold serially diluted SeNPs, HP, and SeNPs@HPto attain final concentrations ranging from 20–10 μg/mL, 2–0.6%, and 10–4.5 μg/mL, respectively in a microtitre plate (MTP; Thermo Fisher Scientific, USA). After incubation at 37°C for 24 h, the MIC values were recorded as the lowest concentration which showed complete inhibition of visible growth. The sub-MIC concentrations of SeNPs, HP, and SeNPs@HP were selected for all further experiments in the present study.

### Growth assay

Effects of SeNPs, HP, and SeNPs@HP on growth of CV12472 and *P*. *aeruginosa* PAO1 were determined using micro-dilution assay. After incubation of LB plates overnight at 37°C, cfu/mL of each bacterium was recorded by counting colonies.

### QS competition assay

Anti-QS assay based on AHL-based competition was performed using the two bio-indicator bacterial strains, CV12472 and *A.tumefaciens*NT1, reported previously elsewhere (Singh B. R. et al., 2015).

### Biofilm formation assays

Anti-biofilm activity of the SeNPs@HP was determined using crystal violate (CV) staining MTP assay. Overnight culture of *P. aeruginosa* PAO1 having OD 1.0 at 600 nm was diluted in fresh LB media (1: 10) containing test samples, aliquoted into the 96-well plate and placed in BOD incubator at 37°C for 24 h without agitation. Formed biofilm at the bottom of plate was stained with using 0.5% aqueous solution of CV (Sigma-Aldrich) for 30 min at 37°C. After washing with DW, bounded CV was eluted in absolute ethanol and read the OD at 545 nm using a microplate reader (BioRad, CA, USA). For normalization of recorded values, the OD_545_ value was stabilized by the OD_595_.

Inhibition of biofilm formation was also analyzed by both fluorescent and light microscopy as described recently (Singh B. R. et al., 2015). Briefly, *P*. *aeruginosa* PAO1 was grown on the cover glasses without and with SeNPs@HP for 24 h. Formed biofilms were stained with SYTO-9 (20 mM) for 15 min at room temperature and analyzed by fluorescent microscopy (Nikon, Japan) at 480 nm (excitation). Biofilms of *P. aeruginosa* PAO1 was also stained with 0.5% of CV solution and observed under light microscopy at 20x magnification.

### Virulence factor assays

*P*. *aeruginosa* PAO1 was grown in LB media in the absence and presence of SeNPs@HP in 100 mL conical flask at rotatory shaker incubator (200 rpm). After incubation of 24 h, the supernatant (SN) was collected through centrifugation of culture broth at 12, 000 rpm for 7 min and used for biochemical assays. An exoprotease activity was estimated using hide power azure (Caballero et al., 2001). Briefly, 50 μL SN was mixed with 150 μL of 10% Hide powder azure (Sigma-Aldrich) which was prepared in a 20 mM Tris/HCl (pH 8.0) buffer consisting of 1 mM CaCl_2_. MTP containing reaction mixture was incubated at 37°C for 60 min. The supernatant was centrifuged at 6,000 rpm for 5 min and transferred in the new MTP. The absorbance of sample was recorded at 595 nm. The elastase activity of the SN was assessed using elastin Congo red (ECR; Sigma-Aldrich) as a substrate.ECR (2.5%) in 140 μL of 10 Mm Tris-HCl buffer (pH 7.2) without EDTA was mixed with 100 μL of the SN (Caballero et al., 2001). After incubation of the reaction mixture at 37°C for 1 day, the absorbance of the SN was measured at 490 nm using a Bio-Rad microplate reader. For quantifying pyocyanin content, the SN (5 mL) was fractionated with chloroform (3 mL) containing trichloroacetic acid (MP, Biomedicals Inc.). To re-extract pyocyanin, fractionated layer was again extracted with 1 mL of 0.2N HCl and recorded absorbance at 520 nm in a UV-vis spectrophotometer (Thermo Fisher Scientific, USA) (Essar et al., 1990). Rhamnolipid content was measured by adjusting the pH of SN (10 mL) with HCl and fractionated twice by mixing 10 mL of diethyl ether. The solvent was evaporated to dry at rotary evaporator (Buchi, USA) and obtained sample was dissolved 10 mL DW. Then, the sample (20 μL) was mixed with 180 μL of 1% orcinal solution, prepared in 15% H_2_SO_4_. The reaction mixture was boiled for 30 min and measured the OD at 421 nm using a Bio-Rad microplate reader (Boles et al., 2005).

### Rats wound healing and mice mortality assays

The wound healing property of SeNPs@HP was assessed using excision wound models (Mughrabi et al., 2011). Swiss albino rats weighing 140 ± 5 g were used for the study. All studies were carried out in accordance with the guidelines of the National Institutes of Health Guide for the Care and Use of Laboratory Animals and all experimental protocols were approved by an Institutional Animal Care Committee, CPCSEA, India (Reg. No. 222/2000/CPCSEA). The rats were anesthetized and their dorsum was shaved. After disinfection of dorsum area with 75% ethanol, an excision wound (4 × 4 cm) was made and then infected with overnight culture of *P*. *aeruginosa* PAO1, having 2 × 10^7^ cfu/wound by injecting the bacterium directly into the wound. Four groups were made and six rats were used for each group (Table 3). The wounds were covered with plain gauze and rats were placed into individual cages. Graph paper was used to measure wound areas and results were expressed in terms of percent wound contraction using the following equation: Wound contraction (%) = [Initial wound size–specific day wound size]/Initial wound size × 100.

Mice mortality test was also performed to examine protective effect of SeNPs@HP. The cell pellet was prepared from overnight culture of *P*. *aeruginosa* PAO1 through centrifugation at 12,000 rpm for 8 min at 4°C. After washing with DW, the pellet was dissolved in PBS and 100 μL cell suspension (2.0 × 10^7^ cfu/mL) was injected into the abdominal cavity of 4 weeks old pathogen free female mice weighing 18 ± 2 g. Mortality was measured during a 55 h period for 12 mice for each treatment. DW was injected into mice as a vehicle control.

### HPLC analysis

For preparing extract, SeNPs@HP was mixed with absolute ethanol for 2 h with constant shaking at 100 rpm. The solvent was removed using rota evaporator at 40 ± 1°C. Presence of phytochemicals was confirmed by Shimadzu LC-10A (Kyoto, Japan) HPLC system (Singh et al., 2010). The separation of compounds was achieved on a solvent phase consisting acetonitrile/water (1:1, v/v) containing 1% acetic acid in a linear gradient program, started with 18% acetonitrile, changing to 32% in 15 min and finally to 50% in 30 min. The compounds were obtained by comparison of peak areas (λmax 254 nm) of the extract with those of standards.

### Isolation of total RNA and RT-PCR analysis

Total RNA was isolated from *P. aeruginosa* PAO1 biofilm cells using the QIAzol Lysis Reagent (QIAGEN) following the manufacturer's instruction. The reaction mixture was prepared by adding 2 μL template RNA, 10 μL SYBR master mix (Thermo Scientific, USA), 0.8 μL each of the forward and reverse primers (10 mM), 0.4 μL reference dye, and water (RNA free) to make up a 20 μL total volume. Analysis was performed at 50°C for 50 min followed by denaturation for 10 s at 95°C, annealing for 10 s for 55°C, and extension for 35 s at 60°C. Eventually, a dissociation analysis (95°C for 15 s, 60°C for 1 min, and 95°C for 15 s) was also carried out to confirm that absence of non-specific amplicons. The obtained cDNA samples were stored at −80°C. The primer sets for the genes were designed using Primer 3 (v 0.4.0) (Supplementary Table 1).

### Computational molecular docking studies

Molecular docking analysis was performed to identify the conformational changes in the protein structure due to the interaction of phytochemicals of honey with LasR receptor protein. Ligand structures were downloaded from Pubchem NCBI in sdf format and all were converted to pdb format using online tool “SMILE CONVERTER” for further docking analysis. The energy was minimized using AutoDock Tools by defining the rotable bond, torsion angle and submerging the non-polar hydrogens. Three-dimensional structure of LasR receptor protein was retrieved from RSCB PDB database (PDB ID: 2UV0). PDB 2UV0 structure comprises four chains (E, F, G, and H) whose confirmation was similar, confirmed by superimposing with chimera. Since, the H chain is longest and contained the preferred binding site for the natural ligand, all the water molecules and other chains were removed from the LasR receptor protein. Docking calculations were carried out using the graphical user interface program “AutoDock Tools.” Kollman united atom charges, solvation parameters and polar hydrogens were added into the receptor PDB files for the preparation of protein in docking simulation. The grid map was assign 70, 70, and 70 A° toward X, Y, and Z axis respectively to include all amino acid of binding site of receptor protein for docking and grid point spacing was 0.375A°. The Lamarckian genetic algorithm (LGA), considered one of the best docking methods available in AutoDock, was adopted to perform the molecular docking studies. Both AutoGrid and AutoDock computations were executed on Cygwin. Final docked conformations were clustered using a tolerance of 2 A° RMSD and the docking log (dlg) files were analyzed using AutoDock Tools, the graphical user interface of AutoDock. The docked conformations of each ligand were ranked into clusters based on the binding energy and the top ranked conformations were visually analyzed. Hydrogen bonding and hydrophobic interactions between docked ligand and macromolecules were analyzed by Discovery Studio software.

## Author contributions

BNS and VG conceived and designed the experiments. PG, BRS, MS, and SS performed most of the experiments. PG, BRS, MS, SS, and AN. analyzed the experimental data. VG and BNS wrote the manuscript.

### Conflict of interest statement

The authors declare that the research was conducted in the absence of any commercial or financial relationships that could be construed as a potential conflict of interest.
