# Supplementary material for: Scaffold of Selenium Nanovectors and Honey Phytochemicals for Inhibition of Pseudomonas aeruginosa Quorum Sensing and Biofilm Formation
Source: Front Cell Infect Microbiol. 2017 Mar 23;7:93. doi: 10.3389/fcimb.2017.00093 (PMC5362927; doi:10.3389/fcimb.2017.00093)
Supplement: Supplementary file 1 [file Presentation1.PDF]

Scaffold of selenium nanovectors and honey phytochemicals for inhibition of *Pseudomonas aeruginosa* quorum sensing and biofilm formation

Prateeksha[1§], B.R. Singh[2§], M. Shoeb[2], S. Sharma[1], A.H. Naqvi[2], V.K. Gupta[3*] and Brahma N. Singh[1*]

[1]Pharmacognosy & Ethnopharmacology Division, CSIR-National Botanical Research Institute, Lucknow-226001, India
[2]TERI-Deakin Nanobiotechnology Centre, The Energy Research Institute Darbari Seth Block, IHC Complex, Lodhi Road, New Delhi-110003, India).
[3]Molecular Glyco-biotechnology Group, Discipline of Biochemistry, School of Natural Sciences, National University of Ireland Galway, Galway, Ireland

Keywords: Selenium; nanovectors; honey; polyphenols; quorum sensing; *Pseudomonas aeruginosa*

[§]These authors are equally contributed

*Corresponding authors:
singhbrahmanand99@gmail.com (B.N. Singh)
vijaifzd@gmail.com (V.K. Gupta)

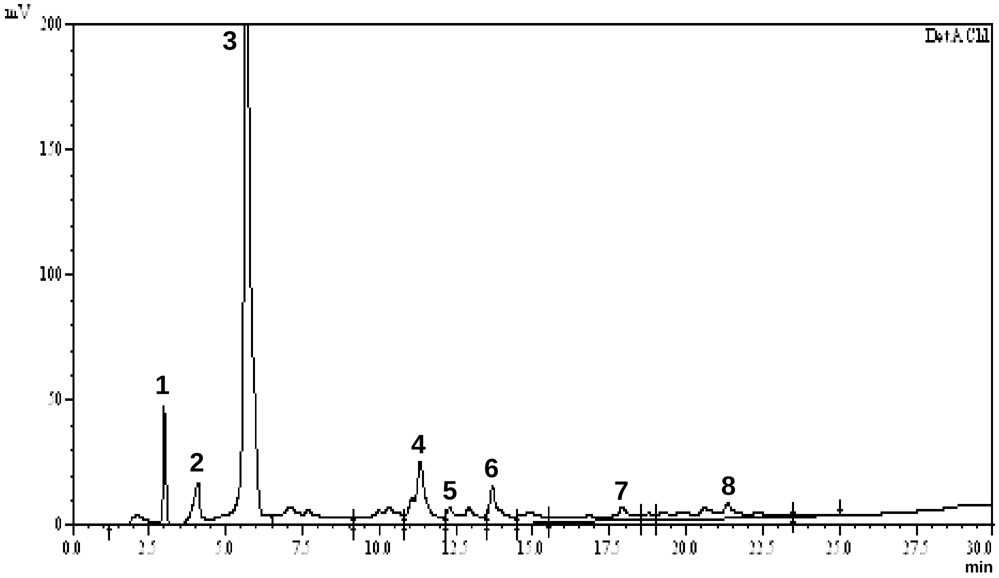

Supplementary figure 1. RP-HPLC fingerprinting. The SeNPs@HP was dissolved in absolute ethanol for 2 h and presence of phytochemicals was confirmed by RP-HPLC.A solvent phase, acetonitrile/water (1:1, v/v) containing1% acetic acid in a linear gradient program was used. Phytochemicals were identified by comparison of peak areas (λmax 254 nm) of the extract with those of standards. HPLC chromatogram shows the presence of chrysin (1), apigenin (2), caffeic acid (3), acacetin (4), quercetin (5), kaempferol (6), pinocembrin (7), and pinobanksin (8).

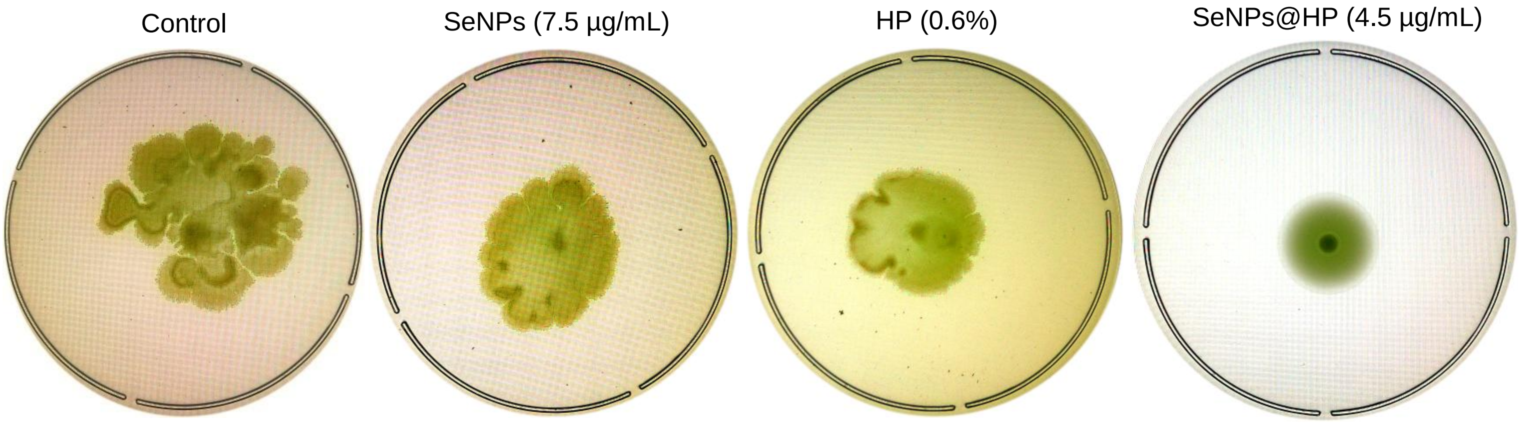

Supplementary figure 2. Effect of SeNPs@HP on swarming motility. *P. aeruginosa* PAO1 was grown on LB media containing 0.3% agar, SeNPs (7.5 µg/mL), HP (0.6%), SeNPs@HP (4.5 µg/mL), and furanone C-30 (10 µM).

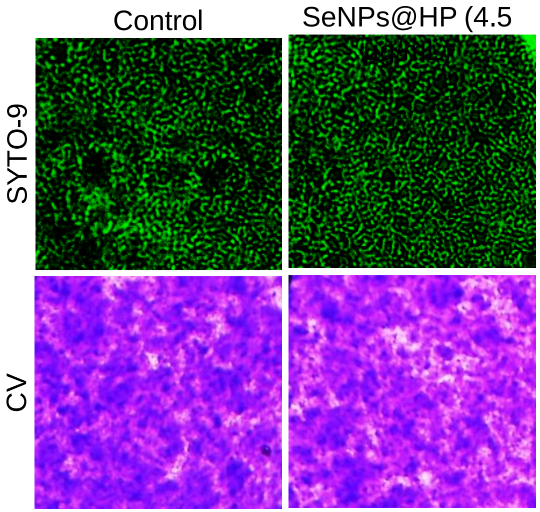

Supplementary figure 3. Effect of SeNPs@HP on biofilm inhibition in *E. coli* transformed by a plasmid overexpressing LasR.

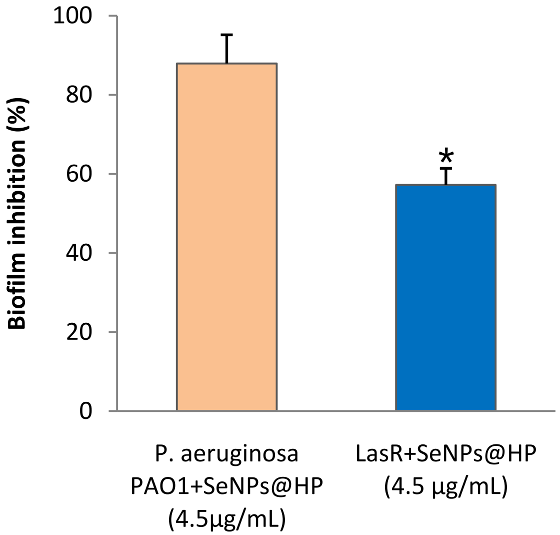

Supplementary figure 4. Effect of SeNPs@HP on biofilm inhibition in P. aeruginosa PAO1 and *E. coli* transformed by a plasmid overexpressing LasR. Error bars indicate the standard deviations of 3 measurements. *, p, <0.01 versus the *P. aeruginosa* PAO1.

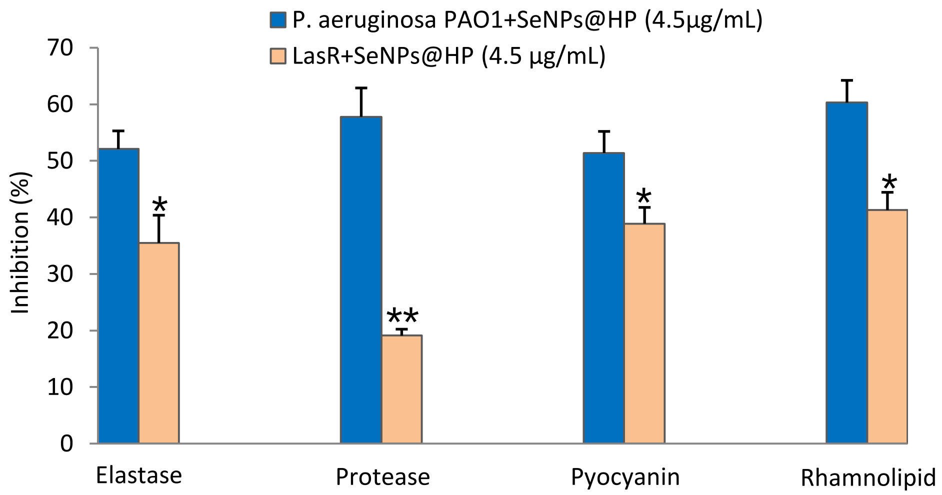

Supplementary figure 5. Effect of SeNPs@HP on production of virulence factors elastase, protease, pyocyanin, and rhamnolipid in P. aeruginosa PAO1 and its LasR mutant . Error bars indicate the standard deviations of 3 measurements. **, p, <0.001 versus the *P. aeruginosa* PAO1. *, p, <0.01 versus the *P. aeruginosa* PAO1.

Supplementary Table 1. List of primers

Gene	Direction	Sequence
lasA	F	GTTCCTCTTCGTCTTGCTGG
	R	GCTCCAGGTATTCGCTCTTG
lasB	F	ACATCGCCCAACTGGTCTAC
	R	CCGTAGGTGTACTTGCCGAT
lasI	F	GGCTGGGACGTTAGTGTCAT
	R	AAAACCTGGGCTTCAGGAGT
lasR	F	GTTTTCTTGAGCTGGAACGC
	R	CGTCTGGTAGATGGACGGTT
rhlA	F	AGCTGGGACGAATACACCAC
	R	GACTCCAGGTCGAGGAAATG
rhlB	F	GAGCGACGAACTGACCTACC
	R	GTTGAACTTGGGGTGTACCG
rhlI	F	CTCTCTGAATCGCTGGAAGG
	R	GATGGTCGAACTGGTCGAAT
rhlR	F	ACCAGATGCAGAACTACGGG
	R	GCTCGAAGCTGGAGATGTTC
rpoS	F	GGACAAGACCCTGCTGGATA
	R	CTCGACAGGCCATTCTTCTC
norC	F	GCATGGCCAGGAACATCTAT
	R	CTGGAGGAAGGTGTTGAAGC
phzA2	F	GGTTTACCGACAACCTGGAA
	R	CACTCGACCCAGAAGTGGTT
phzB1	F	GAACTTCGCGAAAAGAATCG
	R	ATCGGGAAAGCACTTCAATG
phzC1	F	GGATCCTCAAGGGCTATGC
	R	GTGGGTCGAACCGAGATAGA
phzE1	F	GAGGAGCTGAAGATGATGGC
	R	GTCTGGCCCTCGATGAAGTA
proC	F	GAGCAACTGATCGTCTCCATC
	R	TCAGCAGGAAGAAATACGCC
hcnA	F	CATGACCATCCACCTCAATG
	R	GGTTGCTTTCGGTTTCCAC
hcnB	F	GCTGCACGAGGTGGAGTATT
	R	ACAGTTGCGGCTTGTTCAG
hcnC	F	CTTCTTCCGCATGATGTCCT
	R	AACTTGAAGTCCATCCCGTG
chiC	F	TCCGTCTGGTGGAAACCTAC
	R	CATGCTGACGATGAAGTGCT
fabH1	F	GAGTCTCGAAGACGGACTGC
	R	GTTGATGGCAGATCACATGG
fabH2	F	CTTTCAGCCATACCCTCGAC
	R	TCCATCCGGAAGTAGTTGGA
acpP	F	GCGTTAAGAAGATCGTCGCT
	R	TCCTCTTCCAGAGCCATCAC
Psl	F	TCGGTAGATAGCCTGTCGCT
	R	TGAACAACAGCAGGCAGAAC
retS	F	GACTACGTGCAGACCATCCA
	R	CTTCCTCGGTCTGCTTGAAC
